# Preferential Elimination of Older Erythrocytes in Circulation and Depressed Bone Marrow Erythropoietic Activity Contribute to Cadmium Induced Anemia in Mice

**DOI:** 10.1371/journal.pone.0132697

**Published:** 2015-07-10

**Authors:** Sreoshi Chatterjee, Rajiv K. Saxena

**Affiliations:** 1 School of Life Sciences, Jawaharlal Nehru University, New Delhi, India; 2 Faculty of Life Sciences and Biotechnology, South Asian University, New Delhi, India; National Institutes of Health, UNITED STATES

## Abstract

Feeding cadmium chloride (50 or 1000 ppm CdCl2 in drinking water, ad libitum) to C57BL/6 mice resulted in a significant and sustained fall in blood erythrocyte count and hemoglobin levels that started 4 and 3 weeks after the start of 50 and 1000 ppm cadmium doses respectively. A transient yet significant reticulocytosis occurred during the first 4 weeks of cadmium treatment. Using the recently developed double *in vivo* biotinylation (DIB) technique, turnover of erythrocyte cohorts of different age groups was simultaneously monitored in control and cadmium treated mice. A significant accumulation of younger erythrocytes and a concomitant decline in the relative proportions of older erythrocytes in circulation was observed in both 50 and 1000 ppm cadmium groups indicating that older erythrocytes were preferentially eliminated in cadmium induced anemia. A significant increase in the erythropoietin levels in plasma was seen in mice exposed to 1000 ppm cadmium. Levels of inflammatory cytokines (IL1A, IL6, TNFα, IFNγ) were however not significantly altered in cadmium treated mice. A significant increase in cellular levels of reactive oxygen species (ROS) was observed in older erythrocytes in circulation but not in younger erythrocytes. Erythropoietic activity in the bone marrows and spleens of cadmium treated mice was examined by monitoring the relative proportion of cells belonging to the erythroid line of differentiation in these organs. Erythroid cells in bone marrow declined markedly (about 30%) in mice in the 1000 ppm cadmium group but the decline was not significant in the 50 ppm cadmium group. Cells representing various stages of erythroid differentiation in bone marrow and spleen were enumerated flow cytometrically by double staining with anti-Ter119 and anti-transferrin receptor (CD71) monoclonal antibodies. Decline of erythroid cells was essentially confined to pro-erythroblast and erythroblast-A, along with a concurrent increase in the splenic erythroid population indicating a stress response. In short cadmium exposure causes preferential clearance of older erythrocytes from circulation along with a depressed erythropoietic activity at higher doses.

## Introduction

Cadmium is one of the most toxic heavy metals, a common non-biodegradable environmental contaminant that causes serious health hazard [1–4]. Cadmium exposure may occur through contamination in drinking water and food [5], cigarette smoke [6–7], or through occupational exposure during mining or manufacturing of batteries and pigments, electroplating, production of alloys, and smelting [4, 7–9]. Cadmium, a category I carcinogen [1], has a long biological half life (10–30 years), and it accumulates into various organs and tissues causing severe damage, particularly in kidney [10–12] and also in liver, lung, testis, bone, cardiovascular and the immune system [12–13]. Cadmium is genotoxic; it is often associated with gene mutation, DNA strand breaks, disruption of DNA repair, chromosomal damage and epigenetic changes, resulting in altered gene expression [14–15]. Cadmium induced damage may be mediated through oxidative insults caused by increased ROS generation, lipid peroxidation, inhibition/depletion of antioxidant enzymes and inflammation [16–19]. Most severe example of cadmium toxicity was demonstrated by “itai-itai” (ouch-ouch) disease, endemic to the Jinzu river basin of Fuchu-Toyama Prefecture in Japan, characterized by osteomalacia and osteoporosis, renal tubular dysfunction, immune deficiencies and anemia [20–22].

Severe anemia is often found to be associated with cadmium toxicity in exposed population [22–24]. Cadmium is also reported to induce anemia in laboratory animals [25–27]. Cadmium induced anemia is characterized by both inefficient hematopoiesis (iron deficiency and renal anemia) and hemolysis [27]. It is however not clear if all circulating erythrocytes are equally susceptible to the toxic effect of cadmium or whether erythrocytes of a particular age group are preferentially eliminated in cadmium induced anemia.

In the present study, we have developed a mouse model of cadmium induced anemia to assess the age dependent susceptibility of circulating erythrocytes to cadmium in this model. We used a double *in vivo* biotinylation (DIB) technique, recently developed in our laboratory, for identifying and enumerating erythrocyte subpopulations of different age groups [28–33]. Inbred male C57BL/6 mice were given cadmium in their drinking water (a low dose of 50 ppm and a high dose of 1000 ppm of cadmium chloride) and at different time points their circulating erythrocytes were analyzed using the DIB technique. We also examined the effect of cadmium treatment on erythroid differentiation pathway in bone marrow and spleen. For delineating erythroid precursors at different stages of differentiation, an established protocol of double staining with antibodies against glycophorin receptor Ter119 and transferrin receptor (CD71) was used [34–39]. Our results showed that older erythrocytes in circulation were preferentially eliminated in mice treated with cadmium. At higher dose of cadmium, bone marrow erythropoietic activity was also significantly depressed, the effect being primarily on earlier stages of erythroid differentiation.

## Materials and Methods

### Animals

Inbred C57BL/6 male mice (8–12 weeks old, 20–25 g body weight) were used throughout this study. Animals were bred and maintained in microbe free environment in the animal house facility at Jawaharlal Nehru University (JNU), New Delhi or obtained from the National Institute of Nutrition (NIN), Hyderabad. The animals were housed in positive-pressure air conditioned units (25°C, 50% relative humidity) and kept on a 12 h light/dark cycle. Water and mouse chow were provided *ad libitum*. All the experimental protocols were conducted strictly in compliance with the Guidelines notified by the Committee for the Purpose of Control and Supervision on Experiments on Animals (CPCSEA), Ministry of Environment, Forest and Climate Change (CPCSEA guidelines, www.envfor.nic.in/divisions/awd/cpcsea_laboratory.pdf). The study was duly approved by Institutional Animal Ethics Committee (IAEC) of Jawaharlal Nehru University (IAEC Approved Project Code: 5/2010). All mice were randomly assigned to experimental groups. Experiments were designed so as to use the minimum number of mice. For the analysis of blood erythrocytes, 25–30 μl blood samples were taken weekly from tail-vein at indicated time points. For deriving bone marrow and spleen cells, mice were euthanized by CO_2_ asphyxiation before the organs were dissected out. Terminal blood collection for isolation of plasma from mice was performed by cardiac puncture following CO_2_ asphyxiation.

### Reagents and other Supplies

Biotin-X-NHS (N-hydroxysuccinimide ester of biotin) was obtained from Calbiochem (La Jolla, CA, USA) or from Sigma Aldrich (St. Louis, MO, USA). Streptavidin Allophycocyanin (SAv-APC), rat anti-mouse Ter-119-APC, rat anti-mouse CD71-PE, rat anti-mouse CD47-FITC, anti-mouse CD16/CD32 and Annexin V-PE recombinant protein were obtained either from BD Biosciences (San Diego, CA, USA) or from Affymetrix eBioscience (San Diego, CA, USA). Anti-mouse IgG1k-PE, anti-mouse IgG2bk-APC, anti-mouse IgG2bk-FITC and 7-Aminoactinomycin D (7AAD) were procured from Affymetrix eBioscience (San Diego, CA, USA). 5 (and 6) chloromethyl-2, 7 dichloro-dihydrofluorescein diacetate (CM-H_2_DCFDA) was purchased from Molecular Probes (Eugene, OR, USA). Mouse Erythropoietin (EPO) ELISA kit was procured from BioLegend (San Diego, CA, USA) and Multi-Analyte ELISArray kit (mouse Mix-N-Match for inflammatory cytokines IL1A, IL6, TNFα and IFNγ) was purchased from Qiagen (Germantown, MD, USA). Fetal bovine serum was obtained from Hyclone (South Logan, UT). RPMI, HEPES, Dimethylformamide (DMF), and other analytical reagents were from Sigma-Aldrich (India). Cadmium Chloride was procured from Fisher Scientific, India.

### Double *in vivo* Biotinylation (DIB) Technique

*In vivo* biotinylation of circulating erythrocytes was done as described previously [28–31, 33]. The DIB technique is used for tracking age related changes in a cohort of erythrocyte that enters blood circulation within a defined period of time (5 days), from the time of its entrance in blood until the end of its life span [31, 33]. The DIB protocol A [33] involves two steps of biotinylation of circulating erythrocytes by intravenous administration of biotin-X-NHS Ester (BXN), through the tail vein of mice. In the first step of high intensity *in vivo* biotinylation, three daily intravenous (i.v.) injections of biotin (1 mg BXN dissolved in 20 μl of DMF and 250 μl of phosphate buffered saline (PBS)) were given, followed after 5 days, by a low intensity biotinylation with a single lower dose (0.6 mg of BXN dissolved in 12 μl of DMF and 250 μl of PBS). This low intensity biotinylation labels the fresh erythrocytes that were released in the circulation in the 5-day window following the first biotinylation step. At any time point after the second biotinylation step, biotin intensity on circulating erythrocytes could be analyzed by flow cytometry after staining the erythrocytes with streptavidin coupled to an appropriate fluorochrome as described before [28–30]. Biotin^negative^ erythrocytes in circulation would represent fresh erythrocytes released in blood after the second biotinylation step. Biotin^low^ erythrocytes would represent the cohort of erythrocytes released in blood between the first and the second biotinylation steps, and biotin^high^ erythrocytes would represent the population of residual erythrocytes that were present in the blood at the time of first biotinylation step [33]. The principle of DIB protocol A has been summarized in S1 Fig.

To examine the phosphatidylserine (PS) externalization and CD47 expression in young and old erythrocyte populations, DIB protocol B was followed [33]. In this technique the first biotinylation step coincided with the onset of cadmium exposure, followed by a second low dose biotinylation after a period of 22 days. This would allow simultaneous enumeration of PS externalization and CD47 expression on young (<13 days age) and old (>35 days age) erythrocytes at the peak of anemia, i.e., at 5^th^ week time point. The details of this DIB protocol B has been summarized in S2 Fig.

### Cadmium Treatment and Sample Collection

Cadmium chloride was dissolved in drinking water to get 50 or 1000 ppm solutions of cadmium chloride, and given to mice *ad libitum*. Groups of mice were made as follows. Group I, Control receiving regular drinking water without cadmium; Group II, 50 ppm cadmium chloride solution, and Group III, 1000 ppm cadmium chloride solution. Blood samples (25–30 μl) were collected at different time points from the tail vein in PBS containing 5 mM EDTA. Erythrocyte count and hemoglobin levels were estimated by using an electronic hematology particle counter (Melet Scholesing, MSE4 laboratories). Bone marrow (BM) cells were flushed out of femur and tibia using a 25-gauze needle and resuspended in RPMI medium with 10% FBS. Single cell suspensions of spleen cells were made by gently teasing the spleen in a small volume of PBS. Splenic and BM cells were strained through a fine sieve, pelleted by centrifugation, washed and suspended at desired concentration in RPMI with 10% FBS.

### Measurement of Intracellular Reactive Oxygen Species (ROS)

Intercellular ROS levels were assessed as described before [36, 38–39]. Briefly, erythrocytes were washed and resuspended in pre-warmed PBS supplemented with 2% FBS and incubated with CMH_2_DCFDA stain (5 μM) in the dark for 30 minutes at 37°C in an atmosphere of 5% CO_2_ in air. The oxidative conversion of CMH_2_DCFDA to its fluorescent product by ROS was measured immediately by flow cytometry [38–39].

### Measurement of Erythropoietin (EPO) and inflammatory cytokine levels in plasma

Plasma EPO concentration in control and cadmium treated mice was estimated by using a mouse Erythropoietin ELISA kit following the procedure recommended by the manufacturer (BioLegend). To estimate the level of inflammatory cytokines IL1, IL6, TNFα and IFNγ in plasma of control and cadmium treated mice, a customized Multi-Analyte ELISArray kit (Mouse Mix-N-Match) was used as per the manufacturer’s directions (Qiagen).

### Flow Cytometric Analysis

Mouse blood was collected in PBS containing 5 mM EDTA and washed 3 times with ice cold saline containing HEPES buffer (10 mM, pH-7.4) and 1% FBS. Biotin labeled erythrocytes (1×10^6^) were stained with streptavidin-APC and anti-mouse CD71-PE antibody to delineate different age cohorts of circulating erythrocytes including reticulocytes [28, 33, 38–40]. To examine PS externalization these DIB-labeled erythrocytes were co-stained with Annexin-V-PE recombinant protein by the procedure recommended by the manufacturer (BD Biosciences). Briefly, erythrocytes were suspended in Annexin-V binding buffer (10 mM Hepes, pH 7.4, 140 mM NaCl, 2.5 mM CaCl_2_) and incubated with Annexin-V-PE for 20 minutes in dark. To assess the expression of CD47 marker on erythrocyte membrane, DIB labeled erythrocytes were stained with anti-mouse CD47-FITC along with Streptavidin APC and anti-mouse CD71-PE. All the stained cells were washed and analyzed immediately on a flow cytometer. For all the flow cytometric analysis 7AAD was used as viability dye and immunophenotyping was carried out on gated live 7AAD^-ve^ cells. A minimum of 10,000 events were recorded for each sample.

For enumerating erythroid cells at different stages of differentiation in bone marrow and spleen, freshly prepared single cell suspensions from bone marrow or spleen were first incubated with anti-CD16/32 monoclonal antibody (Fc block, 1 μg/10^6^ cells in 50 μL of PBS+2%FBS) for 10 min followed by staining with anti-mouse CD71-PE and anti-mouse Ter-119-APC for 20 minute at 4°C [36–37]. To determine the proportion of erythroid cells undergoing apoptosis, bone marrow and spleen cells were co-stained with 7AAD and Annexin-V-PE along with anti-mouse CD71-FITC and anti-mouse Ter119-APC. Apoptotic cells were identified as 7AAD^-^ Annexin V^+^ Ter119^+^ cells. After incubation, cells were washed and analyzed by flow cytometry. A minimum of 50,000 events were recorded for the erythroid cells in bone marrow and spleen.

All the flow cytometric analyses were performed on a BD FACSCalibur flow cytometer (Becton Dickinson, San Jose, CA, USA) using Cell Quest software or on a BD FACSVerse flow cytometer (Becton Dickinson, San Jose, CA, USA) using FACSuite software for acquisition and analysis.

### Statistical Analysis

Each experiment was repeated at least three times. Statistical analysis by two-way ANOVA and Student’s t-test was carried out by using SigmaPlot software. Data are presented as means ± SEM. A level of *p* < 0.05 was accepted as statistically significant.

## Results

### Cadmium induced anemia in mice

**Fig 1:**
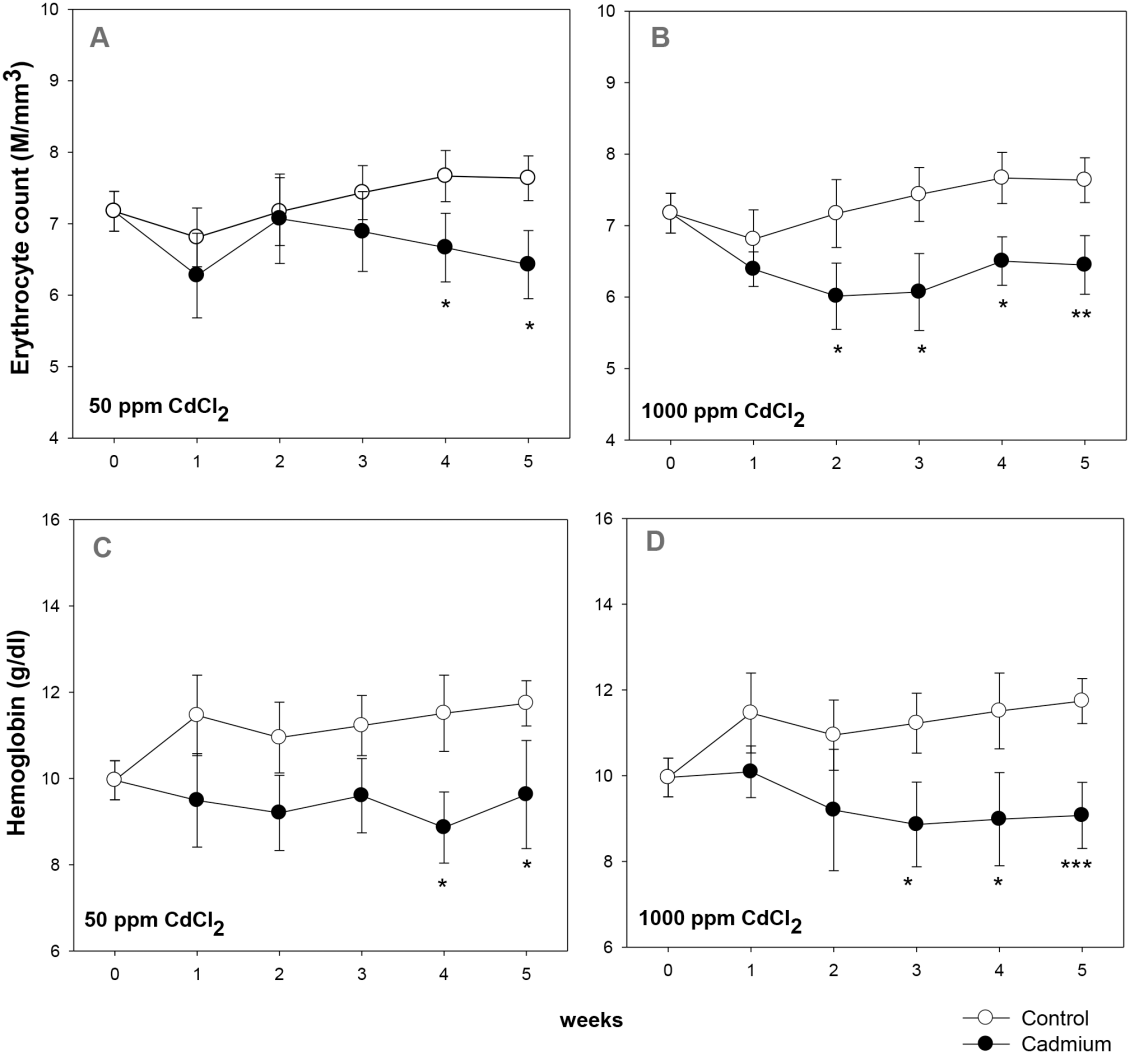
Kinetics of induction of anemia in cadmium treated mice. Mice were given cadmium chloride dissolved in drinking water (50 ppm and 1000 ppm). Blood samples from mice in control and cadmium-treated groups were collected at different time points and analyzed on an automated cell counter. Erythrocyte count and hemoglobin content in 50 ppm group over a period of 6 weeks are given in panels A and C respectively (ANOVA test for effect of cadmium on erythrocyte count, *p* = 0.057; on hemoglobin level *p*<0.001). Erythrocyte count and hemoglobin content at different time intervals in mice exposed to 1000 ppm of cadmium chloride have been shown in panels B and D respectively (ANOVA test for effect of cadmium on erythrocyte count, *p* = 0.001; on hemoglobin level *p*<0.001). Each point on the graph represents mean ± SEM of observations on 10–15 mice. *p<0.05, **p<0.01 and ***p<0.005 for comparison of the groups (Student t-test).

Groups of mice were given 50 ppm (low dose) or 1000 ppm (high dose) of cadmium chloride in drinking water. Blood erythrocyte count and hemoglobin levels were monitored in control and cadmium exposed groups. The results are shown in Fig 1. In the 50 ppm CdCl_2_ group, significant decline in both erythrocyte count as well as in hemoglobin level occurred 4 weeks after the beginning of cadmium exposure (Fig 1A and 1C). In the high dose (1000 ppm) group, however, a significant decline in blood erythrocyte count could be observed as early as in the 2^nd^ week of exposure (Fig 1B), that persisted throughout the treatment schedule. In the 1000 ppm group the erythrocyte count went down from 7.43 ± 0.38 million/mm^3^ in control to 6.07 ± 0.54 million/mm^3^ in the 3^rd^ week, (overall 15%- 18% decline; Fig 1B), while the hemoglobin content fell to 9.07 ± 0.77 g/dl from 11.74 ± 0.53 g/dl in control in the 5^th^ week (overall 21%- 23% decline; Fig 1D).

### Turnover of erythrocytes in cadmium treated mice

**Fig 2:**
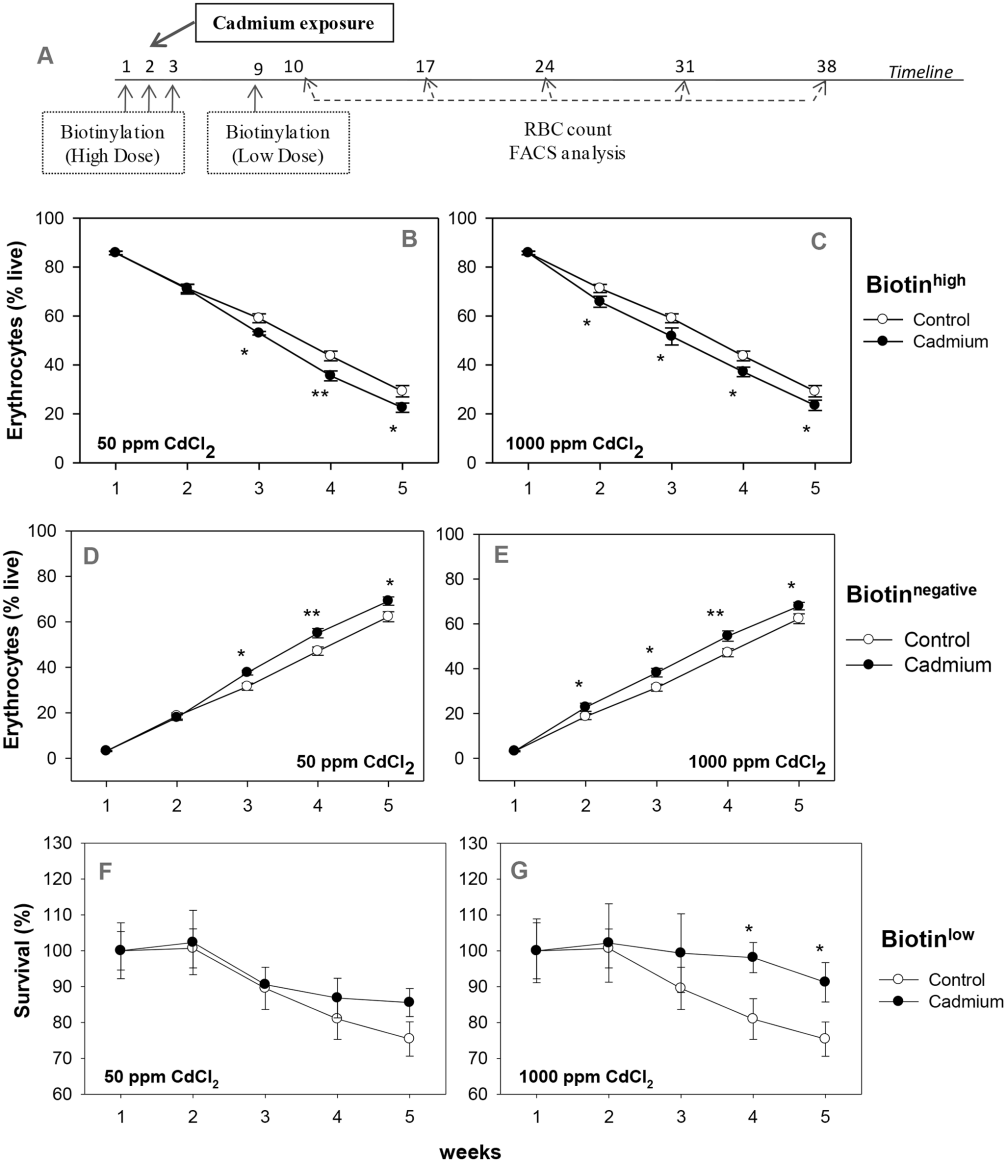
Erythrocyte turnover in the blood of control and cadmium-treated mice. Mice were given cadmium chloride dissolved in drinking water (50 ppm and 1000 ppm). Mouse erythrocytes were labeled with biotin *in vivo* by the two step biotinylation procedure. The experimental protocol is given in panel A. The first biotinylation injection coincided with the onset of exposure of cadmium. After the 2^nd^ biotinylation step, blood samples were collected at weekly intervals. Erythrocytes were stained *ex vivo* with Streptavidin-APC and proportions of the different age cohorts were determined. Turnover profile of biotin^high^ (panels B, C) and biotin^negative^ (panels D, E) erythrocytes in mice exposed to 50 ppm (panels B, D; ANOVA test for cadmium *p* = 0.004 in both biotin^high^ and biotin^negative^ erythrocytes) and 1000 ppm (panels C, E; ANOVA test for cadmium *p*<0.001 in both biotin^high^ and biotin^negative^ erythrocytes) of cadmium chloride have been shown. Relative survival of the biotin^low^ erythrocytes in control and cadmium treated mice is given in panels F and G (for 50 ppm and 1000 ppm of cadmium chloride exposure respectively). For this, proportion of biotin^low^ erythrocytes on the 1^st^ time point in each group was taken as 100 and their proportion at later time points were depicted in relative terms. ANOVA test for effect of cadmium on the relative survival of biotin^low^ erythrocytes in the 3–5 week time point, *p* = 0.32 (not significant) for 50 ppm group (panel F) and *p* = 0.008 for 1000 ppm group (panel G). Each point on the graph represents mean ± SEM of observations on 10–15 mice. *p<0.05 and **p<0.01 for comparison of the groups (Student t-test).

To assess whether the age of erythrocytes in circulation is related to their susceptibility to hemolysis during cadmium induced anemia, the double in vivo biotinylation (DIB) technique of erythrocyte labeling was employed to identify different age groups of circulating erythrocytes [28,33] (concept explained in S1 Fig), and their turnover was studied in both control and the cadmium exposed groups. Experimental protocol showing the beginning of cadmium exposure, the two biotinylation steps and the time points of collection of blood samples is shown in Fig 2A. Erythrocytes isolated from the peripheral blood were stained *ex vivo* with Streptavidin-APC, and proportions of biotin^high^, biotin^low^ and biotin^negative^ populations of erythrocytes were determined by flow cytometry (boxes X, Y and Z respectively; S1B Fig). Comparative analysis of these age cohorts across all the treatment groups at regular intervals revealed their turnover profile (Fig 2).

The kinetics of loss of older erythrocytes (biotin^high^ subpopulations that entered blood circulation before first biotinylation step) indicate that the proportion of older subpopulation of erythrocytes declined at a significantly higher rate in 50 and 1000 ppm cadmium treatment groups as compared to the control group (Fig 2B and 2C). Proportion of younger subpopulation (biotin^negative^, entering blood circulation after the second biotinylation step) increased at a significantly higher rate in both cadmium treatment groups as compared to the control (Fig 2D and 2E). Decline in older (biotin^high^) erythrocyte populations was comparable at both doses of cadmium though the effect was seen earlier (2 weeks) at higher dose than at the low dose (3 weeks) (Fig 2B and 2C). Similarly, the relative increase in younger erythrocyte population in cadmium treated groups were comparable though the increase started 2 weeks post exposure in 1000 ppm group and 3 weeks post exposure in 50 ppm treatment group (Fig 2D and 2E). These results suggest that the older erythrocytes in blood circulation may be preferentially eliminated as compared to the younger erythrocytes in cadmium treated mice.

Survival kinetics of the cohort of biotin^low^ erythrocytes that entered in blood circulation between the first and the second biotinylation steps, showed a lesser decline as compared to control, in the 50 ppm cadmium treatment group 4 and 5 weeks post treatment, indicating that 4–5 week old erythrocytes were less prone to removal in cadmium treated mice (Fig 2F, S1 Table). Better survival of biotin^low^ erythrocyte cohorts that was statistically significant at 3 to 5 week points in 1000 ppm treatment group is clearly seen in Fig 2G.

**Fig 3:**
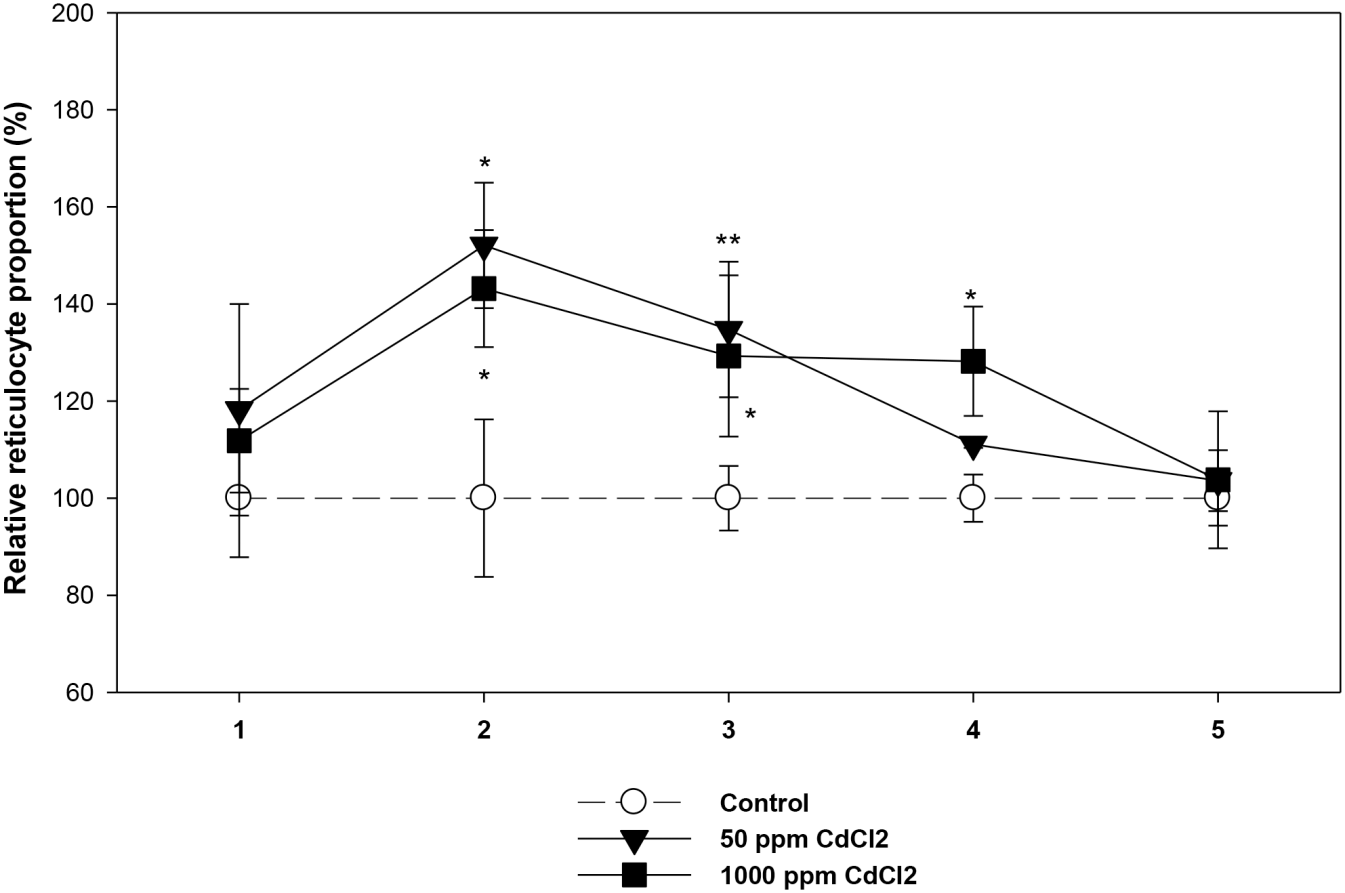
Circulating Reticulocytes in control and cadmium treated mice. Mice were given cadmium chloride dissolved in drinking water (50 ppm and 1000 ppm). At different time points after cadmium exposure, mouse erythrocytes were stained *ex vivo* with anti-mouse CD71-PE and the proportion of circulating reticulocytes were determined by flow cytometry. Reticulocyte count in control ranged between 3.09 to 4.73% (mean = 3.77 ± 0.24). Mean reticulocyte counts in control group of mice at each time point was taken as 100 and the proportion of reticulocytes in the exposed groups were estimated in relative terms. The relative proportions of reticulocytes in 50 ppm and 1000 ppm groups of mice have been shown. Each point on the graph represents mean ± SEM of observations on 7–10 mice. *p<0.05 and **p<0.01 for comparison of the groups. Statistical analysis was done using Student t-test.

An initial surge of reticulocytes in mice exposed to both doses of cadmium was clearly seen at 2 and 3 week time points for the lower dose of cadmium and 2, 3 and 4 week time points for the higher dose of cadmium (Fig 3). In both treatment groups, the reticulocyte surge was lost by the fifth week of cadmium exposure.

### ROS production in erythrocytes from Cadmium exposed mice

**Fig 4:**
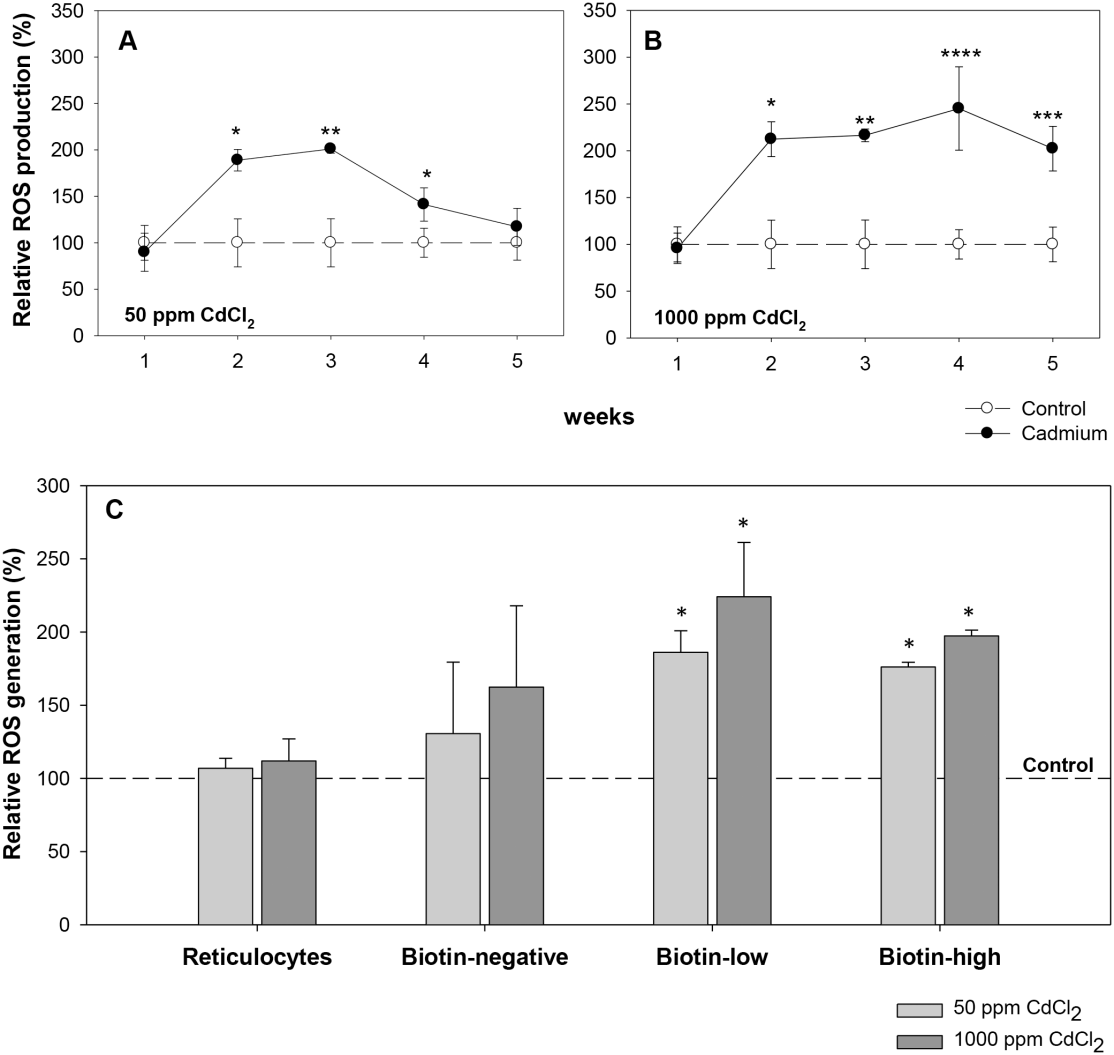
Generation of Reactive Oxygen Species (ROS) in control and cadmium treated mice. Mice were given cadmium chloride dissolved in drinking water (50 ppm and 1000 ppm). Erythrocytes were stained with CMH_2_DCFDA and the ROS generation was determined by flow cytometry. ROS generation in control mice was taken as 100 and the ROS levels in exposed groups were estimated in relative terms Relative ROS generation in mice exposed to 50 ppm (panel A) and 1000 ppm (panel B) of cadmium chloride are shown in the above panels. Mouse erythrocytes labeled *in vivo* by the two step biotinylation procedure were stained with CMH_2_DCFDA, Streptavidin-APC and anti-mouse CD71-PE, and the ROS generation in different subpopulations of erythrocytes was determined. The percent change in ROS generation as compared to control in reticulocytes, biotin^negative^, biotin^low^ and biotin^high^ erythrocytes in control and cadmium exposed mice, after 3 weeks of cadmium exposure have been shown in panel C. Each point and each bar on the graph represents mean ± SEM of observations on 7–10 mice. *p<0.05, **p<0.01 ***p<0.005 and ****p<0.001 for comparison of the groups. Statistical analysis was done using Student t-test.

ROS generation in the erythrocytes of control and cadmium treated groups were estimated by staining erythrocytes with CMH_2_DCFDA. Cadmium exposure resulted in a significant increase in the level of ROS in the whole blood erythrocyte population in both treatment groups (Fig 4A and 4B). In the 50 ppm exposure group, ROS generation increased significantly in the 2^nd^ week of exposure, reached the maximum mean fluorescence intensity (MFI) of 211.61 ± 4.80 from 105.2 ± 27.30 in control, a 2-fold increase) in the 3^rd^ week, and then gradually subsided to normal (control) in the 5^th^ week of exposure (Fig 4A). The 1000 ppm exposure group however showed a consistently increased ROS level, with a maximum MFI of 240.3 ± 43.61 compared to 98.0 ± 15.29 in control (2.5 fold increase) in the 4^th^ week of exposure (Fig 4B).

ROS generation was also examined in erythrocyte cohorts of different ages (reticulocytes, biotin^negative^, biotin^low^ and biotin^high^ erythrocyte populations) in DIB labeled mice exposed to cadmium. ROS generation in different age cohorts of erythrocytes after 3 weeks of exposure is given in Fig 4C. It was observed that in both the low and high dose groups, ROS level had significantly increased (approximately 2-fold) in the biotin^low^ (15–20 days old) and biotin^high^ (>20 days old) subpopulations, though no significant increase was seen in the young (biotin^negative^ and reticulocyte) population. Thus results suggest that the relatively older subpopulations of erythrocytes (biotin^low^ and biotin^high^) may generate more ROS in response to cadmium exposure, which might be a contributing factor in their higher rate of elimination.

### PS externalization and CD47 expression in erythrocytes from Cadmium exposed mice

PS externalization on erythrocyte membrane upon exposure to cadmium was estimated by Annexin-V staining. DIB labeled erythrocytes (DIB protocol B; S2 Fig) were stained with Annexin-V-PE along with Streptavidin APC and anti-mouse CD71-FITC to assess the extent of PS externalization in different age cohorts of erythrocytes in presence of cadmium. Results suggest that upon exposure to high dose of cadmium, PS externalization on erythrocyte membrane increased significantly in the aged cohorts of erythrocytes. After 5 weeks of 1000 ppm CdCl_2_ exposure, the proportion of PS^+^ cells in the aged biotin^high^ erythrocytes (>35 days old) was 0.50 ± 0.07 (% live), almost 2-fold more (*p<*0.01) than the 0.27 ± 0.04 (% live) in young biotin^negative^ erythrocytes (<13 days old), while the same in control remained more or less unchanged. It should however be mentioned that the PS externalization values are very low and would not account for erythrocytes with high PS expression that were immediately phagocytosed *in vivo*.

**Fig 5:**
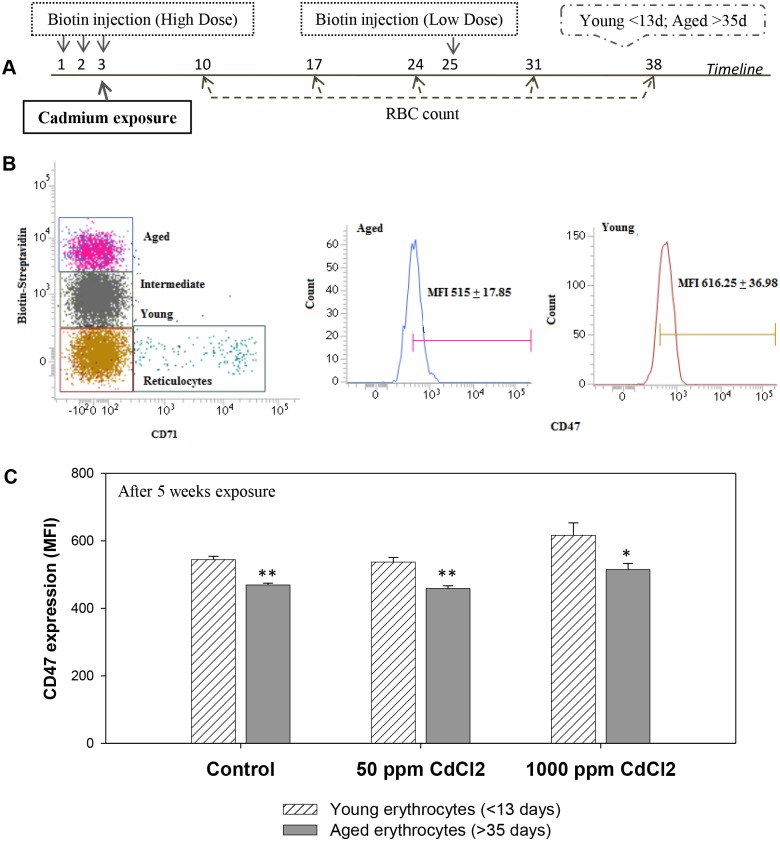
CD47 expression in different age cohorts of erythrocytes. Mice were given cadmium chloride dissolved in drinking water (50 ppm and 1000 ppm). Mouse erythrocytes were labeled with biotin *in vivo* by the two step biotinylation procedure. The experimental protocol is given in panel A. After 13 days of the 2^nd^ biotinylation step, erythrocytes were collected and stained with Streptavidin-APC and anti-mouse CD47-FITC antibody. Representative histograms showing CD47 expression in young and aged erythrocytes is given in panel B. Expression of CD47 on erythrocyte cell membrane among the young (<13 days old) and aged (>35 days old) erythrocytes are shown in panel C. Each bar in the graph represents mean ± SEM of observations on 5 mice. *p<0.005 and **p<0.001 for comparison of the groups (Student t-test).

Erythrocytes of different age groups were also analyzed for the expression of CD47 (“eat me not” signal) on their membrane, by using DIB protocol B. As with earlier reports [29], this study also revealed that in control as well as cadmium treated mice, CD47 expression was significantly lower in older erythrocytes as compared to their younger counterparts (Fig 5).

### Plasma EPO concentration and the level of inflammatory cytokines

**Fig 6:**
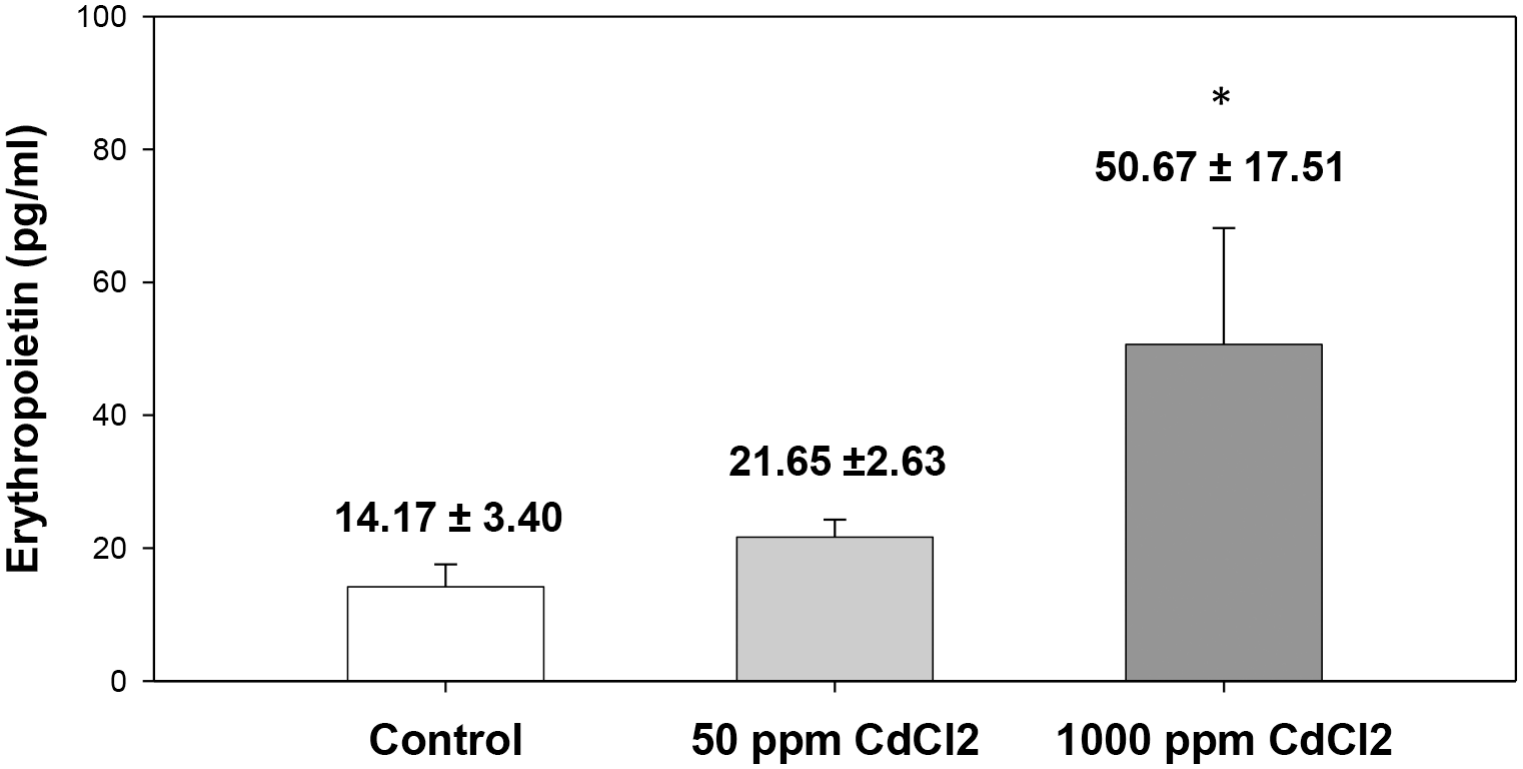
Plasma Erythropoietin (EPO) level in control and cadmium treated mice. Mice were given cadmium chloride dissolved in drinking water (50 ppm and 1000 ppm). After 5 weeks of exposure blood was collected by terminal bleeding and plasma was isolated. EPO levels in plasma were detected by EPO ELISA kit, and the results are shown above. Each bar in the graph represents mean ± SEM of observations on 5 mice. *p<0.05 for comparison of the groups (Student t-test).

Mice were sacrificed and plasma EPO concentration was determined using an EPO ELISA kit. Results depict that after 5 weeks of exposure to cadmium, the plasma EPO levels in cadmium-exposed mice increases almost 4-fold in the 1000 ppm group (Fig 6).

Plasma from the control and cadmium exposed mice were also tested for the presence of inflammatory cytokines like IL1A, IL6, TNFα and IFNγ using a Multi-Analyte ELISArray kit. No significant difference in the plasma levels of IL1a, IL6, TNFα and IFNγ were seen in cadmium treated mice (S2 Table).

### Erythropoietic activity in cadmium exposed mice

**Fig 7:**
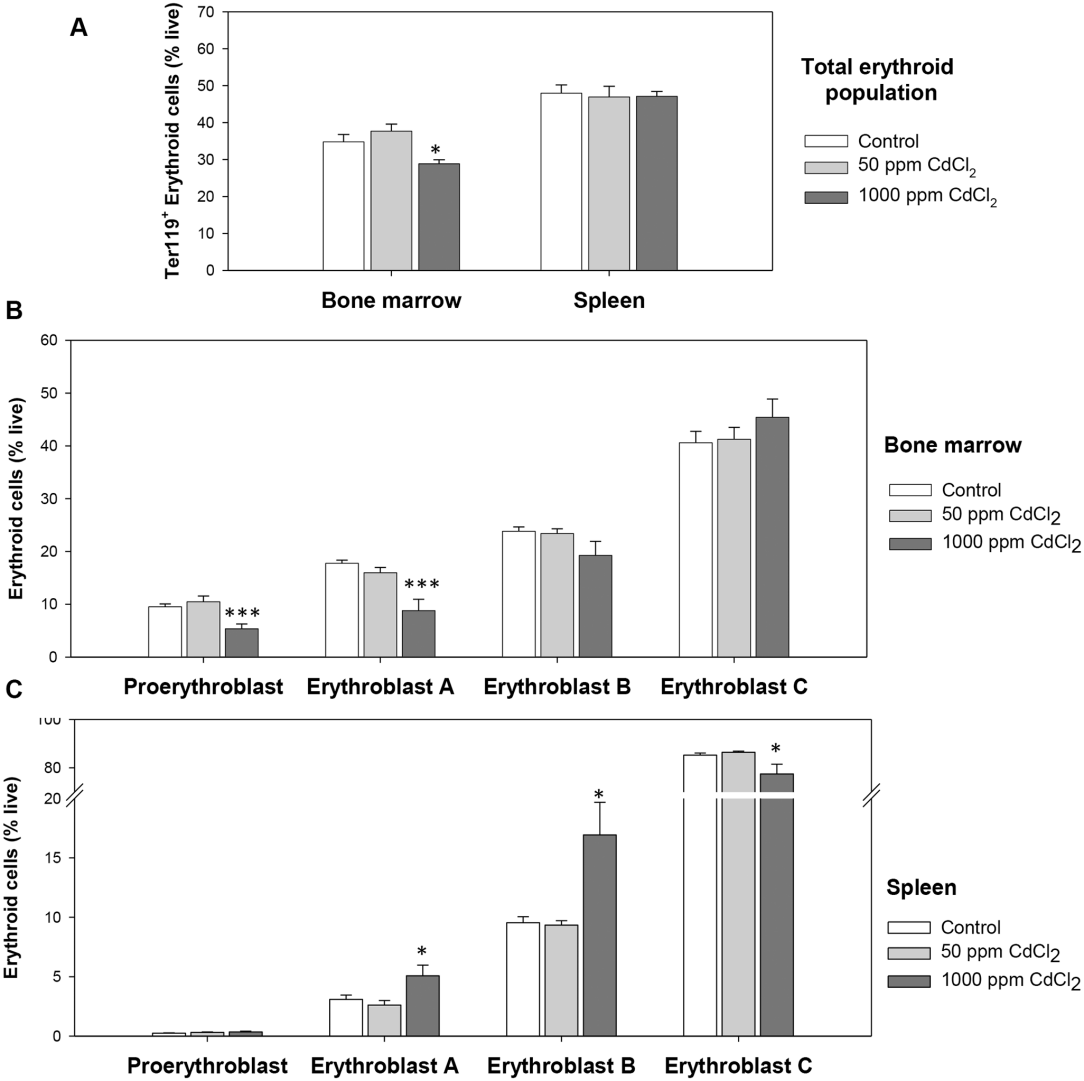
Erythroid population in bone marrow and spleen. Mice were given cadmium chloride dissolved in drinking water (50 ppm and 1000 ppm). Mice were sacrificed and their bone marrow and spleen cells were harvested. Isolated cells were double stained with anti-mouse CD71-PE and anti-mouse Ter119-APC and proportion of cells in different stages of erythropoiesis were determined. Four distinct stages of erythroid differentiation could be identified within an inverted ‘L’ shaped gate in the flow diagram. These include early proerythroblasts (CD71^high^Ter119^med^), early basophilic erythroblast or erythroblasts A (Ter119^high^CD71^high^FSC^high^), late basophilic polychromatic and orthochromatic erythroblasts or erythroblasts B (Ter119^high^CD71^high^FSC^low^), and orthochromatic erythroblasts or erythroblasts C (Ter119^high^CD71^low^FSC^low^). Total erythroid population (panel A), and erythroid cells in different maturational stages in bone marrow (panel B) and spleen (panel C) after 5 weeks of cadmium exposure have been shown above. Each bar in the graph represents mean ± SEM of observations on 5–6 mice. *p<0.05 and ***p<0.005 for comparison of the groups (Student t-test).

Bone marrow and spleen are the two prime erythropoietic sites in adult mice. Possible modulatory effect of cadmium exposure on precursor cell subpopulations at different stages of erythroid differentiation in bone marrow and spleen was examined. Expression levels of Ter119 and CD71 molecules on erythroid cells along with their FSCs in bone marrow and spleen were used to delineate four distinct stages of erythroid differentiation: early pro-erythroblast (Ter119^med^ CD71^high^), early basophilic erythroblast (Ter119^high^ CD71^high^ FSC^high^, erythroblast A), late basophilic, polychromatic and orthochromatic erythroblast (Ter119^high^ CD71^high^FSC^low^, erythroblast B) and orthochromatic erythroblast with mature erythrocytes (Ter119^high^ CD71^low^FSC^low^, erythroblast C), as described elsewhere [35]. A comparison of the relative proportions of erythroid precursor cells delineated within the inverted-L in the flow cytometric histogram fell significantly from 34.78 ± 2.01 to 28.87 ± 1.09 (% live) in the 1000 ppm group after 5 weeks of exposure (Fig 7A). However, no significant effect on the proportion of spleen erythroid cells occurred in cadmium exposed mice (Fig 7A).

**Fig 8:**
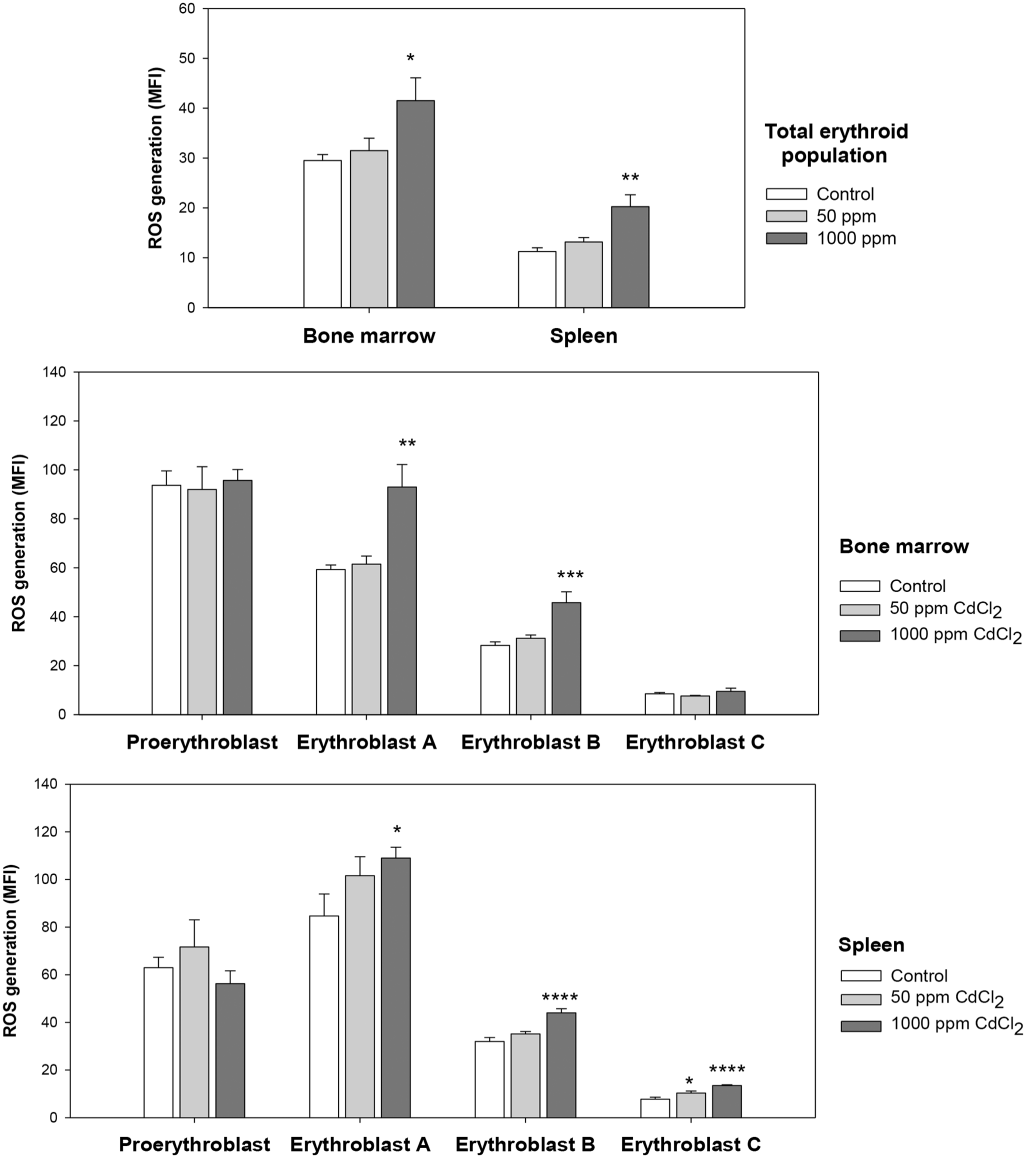
ROS generation in maturational stages of erythroid differentiation in bone marrow and spleen. Mice were given cadmium chloride dissolved in drinking water (50 ppm and 1000 ppm). Mice were sacrificed and their bone marrow and spleen cells were harvested. Cells isolated from mice bone marrow and spleen were stained with PE-anti-mouse CD71, APC-anti-mouse Ter119 and CMH_2_DCFDA. Different maturational stages of erythropoiesis were determined by relative Ter119/CD71/FSC expression, and the ROS generation in each of these stages was estimated by CMH_2_DCFDA staining. Relative ROS generation has been shown as mean fluorescence intensity (MFI) in total erythroid population (panel A) and in different maturational stages of erythroid differentiation in bone marrow (panel B) and spleen (panel C) in mice after 5 weeks of cadmium exposure. Each bar in the graph represents mean ± SEM of observations on 5 mice. *p<0.05, **p<0.01, ***p<0.005 and ****p<0.001 for comparison of the groups. Statistical analysis was done using Student t-test.

A detail analysis of relative proportions of erythroid populations at different stages of erythroid differentiation in bone marrow was done to further analyze the effect of cadmium exposure. Representative flow cytometric data illustrating the gating strategy is given in the S3 Fig. Composite data derived from control and cadmium treated groups of 5 mice each is shown in Fig 7B and 7C for bone marrow and spleen erythroid cells respectively. These results show that at higher dose, cadmium treatment caused a marked and significant decrease in the populations of early stages of erythroid maturation, proerythroblasts and erythroblasts A (around 50% decline; Fig 7B). Analysis of erythroid populations in spleen however revealed a significant increase in the proportions of early stages of erythroid differentiation, indicative of a stress erythropoiesis (Fig 7C). Proportions of Erythroblast A and Erythroblast B showed the maximum increase, >1.5 fold of that in control. This increase in early stages was however stunted by a significant decline in the proportion of the Erythroblast C. To further analyze the cadmium stress acting upon these erythroid cells we studied ROS generation and apoptotic response in erythroid cells in control and cadmium treated mice. ROS generation in the total erythroid population showed a marked increase in mice with 5 weeks of 1000 ppm cadmium exposure (Fig 8A). Bone marrow erythroid cells showed an increase of 40% in mean fluorescence intensity (MFI) as compared to control; while in splenic cells ROS generation went up to 20.25 ± 2.39 from 11.25 ± 0.75 in control (almost 2-fold increase). The lower dose of exposure did not generate any significant ROS. A detail study involving erythroid cells at different stages of maturation revealed significant increase in intracellular ROS levels in the 1000 ppm group in erythroblasts A and B in bone marrow (Fig 8B) and erythroblasts A, B and C in spleen (Fig 8C).

**Fig 9:**
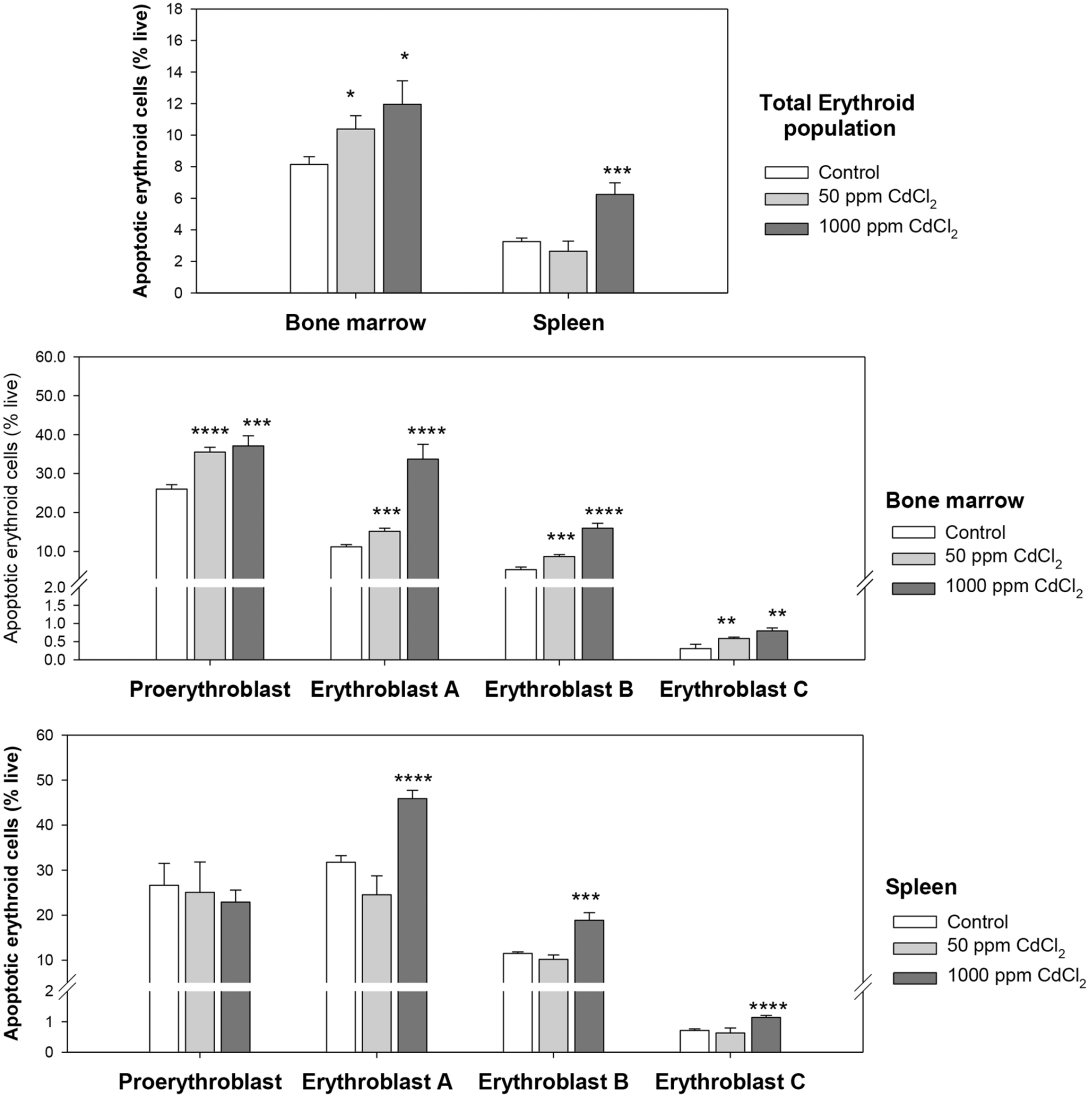
Apoptotic erythroid cells at different stages of maturation in bone marrow and spleen. Mice were given cadmium chloride dissolved in drinking water (50 ppm and 1000 ppm). After 5 weeks of exposure mice were sacrificed and their bone marrow and spleen cells were harvested. Cells thus isolated were stained with anti-mouse CD71-FITC, anti-mouse Ter119-APC and Annexin-V-PE. Different maturational stages of erythropoiesis were determined by relative Ter119/CD71/FSC expression and the apoptotic cells in each of these stages was estimated by Annexin-V staining. Extent of apoptosis in the total erythroid population (panel A) and at different maturational stages of erythroid differentiation in bone marrow (panel B) and spleen (panel C) is shown above. Each bar in the graph represents mean ± SEM of observations on 5 mice. *p<0.05, **p<0.01, ***p<0.005 and ****p<0.001 for comparison of the groups. Statistical analysis was done using Student t-test.

Erythroid cells in bone marrow and spleen were also analyzed for apoptotic response. Annexin-V staining in the total erythroid population in bone marrow and spleen revealed a marked increase (1.5-2-fold) in apoptotic cells (Annexin-V^+^7AAD^-^) after 5weeks of exposure to 1000 ppm CdCl_2_ (Fig 9A). The lower exposure of 50 ppm also resulted in significant increase in apoptosis in bone marrow erythroid progeny, but not in splenic cells (Fig 9A). In bone marrow the proportion of cells undergoing apoptosis increased from 8.14 ± 0.49% in control to 10.39 ± 0.85% in 50 ppm group, and 11.96 ± 1.48% in the 1000 ppm group. In spleen erythroid cells from mice exposed to 1000 ppm CdCl_2_ revealed 6.24 ± 0.74% apoptotic cells as compared to 3.25 ± 0.22% cells in control. A detailed analysis revealed significant increase in the proportion of apoptotic cells at all the stages erythroid maturation in bone marrow in both 50 ppm and 1000 ppm group (Fig 9B). In the higher exposure group maximum effect was observed in erythroblast A and B populations showing a 3-fold increase in apoptotic cells; the 50 ppm group revealed approximately 1.5-fold increase all through. The splenic erythroid cells showed no change in apoptotic response in the lower exposure group, but upon exposure to 1000 ppm CdCl_2_ for 5 weeks, 40–60% increase in Annexin-V^+^ cells were observed in Erythroblasts A, B and C (Fig 9C).

## Discussion

Anemia as a consequence of cadmium toxicity has been observed in many cases of human exposure [22–24], and also in animal models [25–27]. Horiguchi *et al*. [27] have identified three interdependent mechanisms underlying cadmium induced anemia. These include hemolysis of peripheral erythrocytes [41], iron deficiency through inefficient duodenal iron absorption leading to inhibition of heme synthesis [42–43], and renal anemia characterized by hypoproduction of erythropoietin [22–23, 44]. Hemolysis particularly is of interest since majority of the circulating cadmium in blood is found to be concentrated in the erythrocytes [45]. Thus cadmium may induce irreparable deformities in these erythrocytes, accentuating their removal from circulation. It is however not known if the susceptibility of circulating erythrocytes to cadmium is related to their age in circulation.

In the present study we have used a lower (50 ppm), as well as a higher (1000 ppm) dose of cadmium chloride dissolved in drinking water and fed ad libitum to groups of mice. In both the groups cadmium exposure resulted in a significant decrease in the erythrocyte count and hemoglobin content indicating anemia. The anemia was more pronounced in higher exposure group (Fig 1B and 1D), indicating a dose dependent toxicity of cadmium.

For gaining an insight into a possible relationship between the age of erythrocytes in circulation to their susceptibility to elimination in cadmium exposed mice, we used the established technique of double *in vivo* biotinylation (DIB), that has recently been developed in our laboratory and allows us to study the turnover of erythrocyte cohorts of defined age groups in blood circulation [28–32]. Using DIB technique, normal kinetics of age dependent elimination of circulating erythrocytes in mouse has been reported before [33]. It was found that the freshly released erythrocytes in blood remain stable for an initial phase of 10–12 days. This is followed initially by a slower rate of erythrocyte elimination that lasts until 40 days of age in circulation and subsequently an enhanced rate of elimination until all erythrocytes are lost from blood by day 60 [33]. In the current study we found that the kinetics of elimination of erythrocytes in mice exposed to cadmium in drinking water differs significantly from the control pattern of erythrocyte survival. A significant increase in the proportion of the younger biotin^negative^ erythrocytes was consistently observed in cadmium treated mice indicating either an increased generation of fresh erythrocytes or prolonged life span of younger erythrocytes in blood circulation. A surge of reticulocytes in cadmium treated mice (Fig 3) would support the suggestion of more fresh erythrocytes entering the blood. Surge of reticulocytes is a common feature of different types of anemia and may be a consequence of higher plasma levels of erythropoietin that we have shown. Further, a comparison of patterns of elimination of erythrocytes in control and cadmium exposed mice show that younger erythrocytes tend to linger on in circulation for a longer time in the blood in cadmium exposed mice (Fig 2G). The proportion of biotin^high^ old erythrocytes in circulation was significantly lower in the cadmium treatment group, for both high and low doses of cadmium (Fig 2B and 2C), that suggests that older erythrocytes may be preferentially eliminated from the circulation of cadmium treated mice.

Cadmium, though not redox-active, is known to induce its toxicity through oxidative stress both *in vitro* and *in vivo*, and ROS have been considered a crucial mediator for cadmium triggered tissue injury [18–19]. Cadmium cannot generate ROS by itself, but induces depletion and inhibition of antioxidant enzymes such as catalase, superoxide dismutase, glutathione reductase, and glutathione peroxidase [17, 46–47] by binding with sulfhydryl groups in their active sites or by displacing metal cofactors from these active sites [48]. Cadmium may also interfere with the antioxidative stress responses by binding to metallothioneins [49]. We found that cadmium exposure resulted in a significant increase in the level of ROS in the whole blood erythrocyte population in both 50 ppm and 1000 ppm groups (Fig 4A and 4B). In the lower exposure group the increased ROS levels were transient and were normalized by 5^th^ week of treatment (Fig 4A). In the higher cadmium exposure group, consistently high ROS levels could be observed until week 5 (Fig 4B). Examination of ROS levels in different age groups of circulating erythrocytes reveal accumulation of more ROS in older biotin^low^ and biotin^high^ subpopulations of erythrocytes, though not in young biotin^negative^ erythrocytes and reticulocytes (Fig 4C). Higher accumulation of ROS, thus enhanced oxidative stress in the aged subpopulations of erythrocytes (biotin^low^ and biotin^high^), could be one of the contributing factors making aged erythrocytes more prone to destruction. We considered the possibilities of increased ROS being a consequence of a generalized inflammatory response induced by cadmium, but this possibility appears unlikely since levels of inflammatory cytokines (IL1A, IL6, TNFα and IFNγ) remained unaltered in cadmium exposed mice.

Examination of bone marrow and spleen for erythropoietic activity indicated that there was a significant suppression of bone marrow erythropoiesis that declined by about 30% in mice drinking 1000 ppm cadmium chloride as compared to control (Fig 7A). Since significant anemia occurred at both low and high doses of cadmium, it is possible that at lower dose of cadmium the anemia may be due to hemolysis of erythrocytes whereas at higher dose of cadmium, erythropoietic activity in bone marrow may also become a factor contributing to anemia.

Interestingly, unlike in bone marrow, the proportion cells in erythroid line of differentiation in spleen did not decline (Fig 7), instead showed an increase in the proportion of early stages of differentiation (Fig 7B). In some other models of anemia where a depression in bone marrow erythropoiesis occurs, a concomitant increase in spleen erythropoiesis has been documented (stress erythropoiesis) [39–40, 50]. Interestingly, enhanced apoptotic response was observed in erythroid cells from bone marrow as well as in spleen from cadmium treated mice, though it was more pronounced in bone marrow.

Many factors are likely to play a role in cadmium induced anemia. These include the ROS levels in circulating erythrocytes and erythroid cells in bone marrow and spleen. Enhanced reticulocytosis that may be a consequence of a surge of erythropoietin is another factor as is the apoptotic response in cells belonging to the erythroid lineage of differentiation. Apoptotic response in circulating erythrocyte is another possible factor but we found only marginal increase in PS externalization in older erythrocyte population in cadmium treated mice. It is however possible that PS expressing apoptotic erythrocytes may be rapidly removed from circulation by phagocytosis, resulting in only a marginal observable increase in PS expressing erythrocytes in the blood circulation of cadmium treated mice. While our study points to significant changes in some but not all of these parameters, the complex interplay of these factors resulting in anemia remains to be further elucidated.

## Supporting Information

S1 Fig Double in vivo biotinylation (DIB) technique for tracking age related changes on circulating erythrocytes: DIB Protocol A. C57BL/6 mice were administered intravenously three daily doses of 1mg BXN (first biotinylation step). After a rest for five days, a single additional dose of 0.6mg BXN was administered (second biotinylation step). Blood was collected at different time points and distribution of biotin label on erythrocytes was examined by staining the cells with Streptavidin-APC followed by flow cytometry. The scheme of the experiment is given in panel A, and Biotin label on circulating erythrocytes at different time points is given in panel B. Erythrocyte populations in boxes X, Y and Z represent biotin^high^, biotin^low^ and biotin^negative^ populations of erythrocytes respectively; values in parentheses represent percentage of cells in different boxes.
(TIF)

S2 Fig Double in vivo biotinylation (DIB) technique for tracking age related changes on circulating erythrocytes: DIB Protocol B. C57BL/6 mice were administered intravenously three daily doses of 1mg BXN (first biotinylation step). After a rest for 22 days, a single additional dose of 0.6mg BXN was administered (second biotinylation step). Blood was collected at different time points and distribution of biotin label on erythrocytes was examined by staining the cells with Streptavidin-APC followed by flow cytometry. The scheme of the experiment is given in panel A, and Biotin label on circulating erythrocytes at different time points is given in panel B. Erythrocyte populations in boxes X, Y and Z represent biotin^high^, biotin^low^ and biotin^negative^ populations of erythrocytes respectively; values in parentheses represent percentage of cells in different boxes.
(TIF)

S3 Fig Gating strategy for the estimation of erythroid cells of bone marrow at different stages of maturation and the proportion of apoptotic cells amongst them. Mice were sacrificed and their femur and tibia-fibula dissected out. Bone marrow cells were isolated from these bones and single cell suspensions were prepared. 1x10^6^ cells from freshly prepared bone marrow suspensions were stained with Ter119-APC, CD71-FITC, Annexin-V-PE and 7AAD. A representative flow diagram from the bone marrow of a control mouse with all the gating strategies has been shown above. Bone marrow cells were gated on 7AAD^-^ population and the erythroid cells were denoted as the Ter119^+^ population. The Ter119^med^CD71^high^ population amongst these erythroid cells were identified as Proerythroblasts. The remaining Ter119^+^ erythroid cells were further delineated by relative CD71 expression and their FSC and the different stages of maturation were identified as: Erythroblasts A (Ter119^high^CD71^high^FSC^high^), Erythroblasts B (Ter119^high^CD71^high^FSC^low^), and Erythroblasts C (Ter119^high^CD71^low^FSC^low^). Annexin-V^+^ cells amongst each of these erythroid populations indicate the apoptotic cells of erythroid lineage.
(TIF)

S1 Table Turnover of biotin^low^ erythrocytes in control and cadmium treated mice. Mice were given cadmium dissolved in drinking water (50 ppm and 1000 ppm). Mouse erythrocytes were labeled with biotin *in vivo* by the two step biotinylation procedure following DIB protocol A. At weekly intervals erythrocytes were stained *ex vivo* with Streptavidin-APC and proportions of the different age cohorts were determined. Turnover profile of biotin^low^ erythrocytes in mice exposed to 50 ppm and 1000 ppm of cadmium chloride is given in the above table. Each value represents mean ± SEM of observations on 10–15 mice. *p<0.05 for comparison of the groups. Statistical analysis was done using Student t-test.
(TIF)

S2 Table Inflammatory cytokines in plasma of control and cadmium treated mice. Mice were given cadmium chloride dissolved in drinking water (50 ppm and 1000 ppm). After 5 weeks of exposure blood was collected by terminal bleeding and plasma was isolated. Presences of inflammatory cytokines such as IL1A, IL6, TNFα and IFNγ in plasma were detected by a Multi-Analyte ELISArray kit, and the results are given above. Each value represents mean ± SEM of observations on 5–6 mice.
(TIF)
