# Supplementary figures and images for: Preferential Elimination of Older Erythrocytes in Circulation and Depressed Bone Marrow Erythropoietic Activity Contribute to Cadmium Induced Anemia in Mice
Source: PLoS One. 2015 Jul 10;10(7):e0132697. doi: 10.1371/journal.pone.0132697 (PMC4498763; doi:10.1371/journal.pone.0132697)

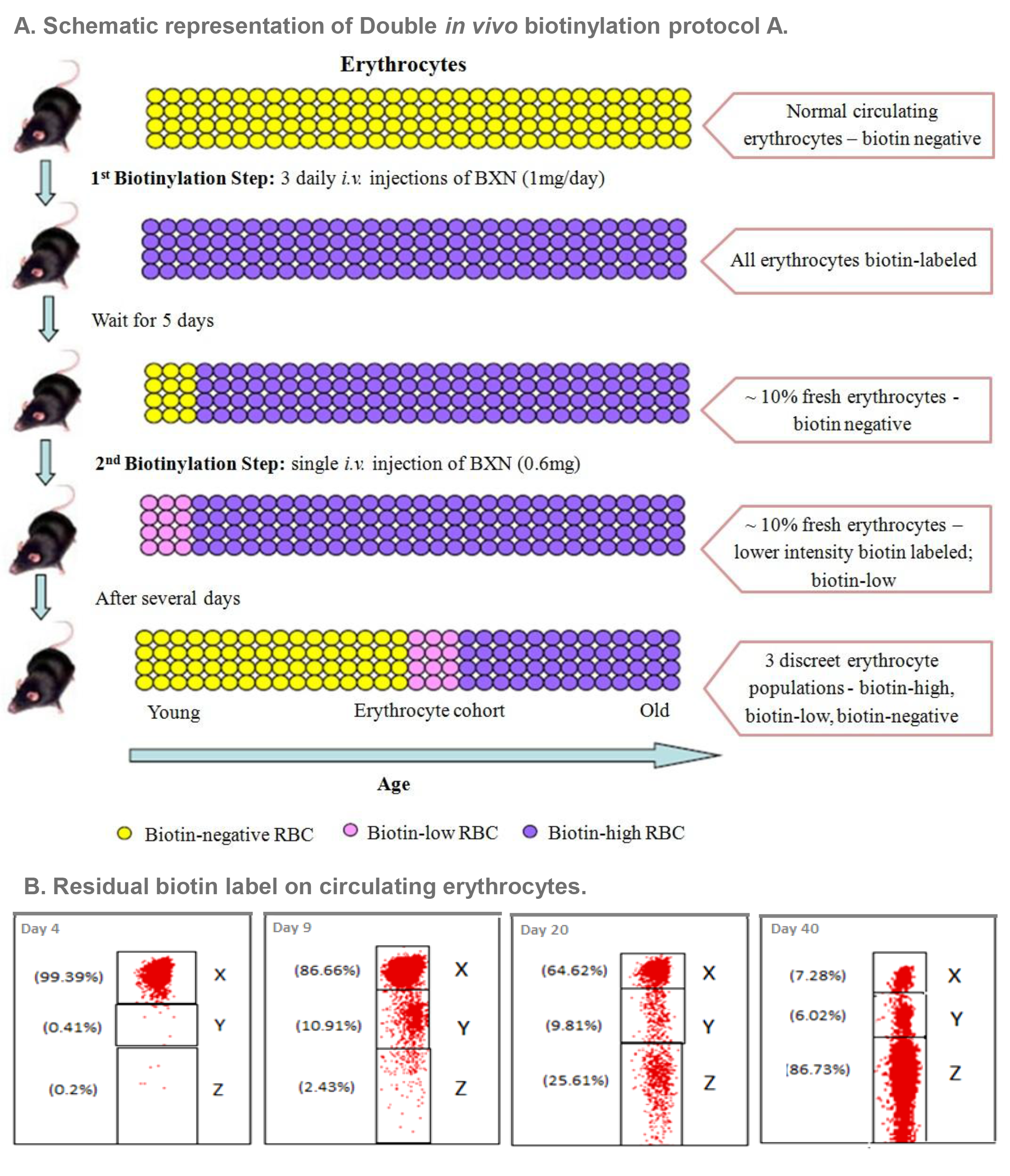

Supplement: S1 Fig — C57BL/6 mice were administered intravenously three daily doses of 1mg BXN (first biotinylation step). After a rest for five days, a single additional dose of 0.6mg BXN was administered (second biotinylation step). Blood was collected at different time points and distribution of biotin label on erythrocytes was examined by staining the cells with Streptavidin-APC followed by flow cytometry. The scheme of the experiment is given in panel A, and Biotin label on circulating erythrocytes at different time points is given in panel B. Erythrocyte populations in boxes X, Y and Z represent biotinhigh, biotinlow and biotinnegative populations of erythrocytes respectively; values in parentheses represent percentage of cells in different boxes. (TIF) [file pone.0132697.s001.tif]

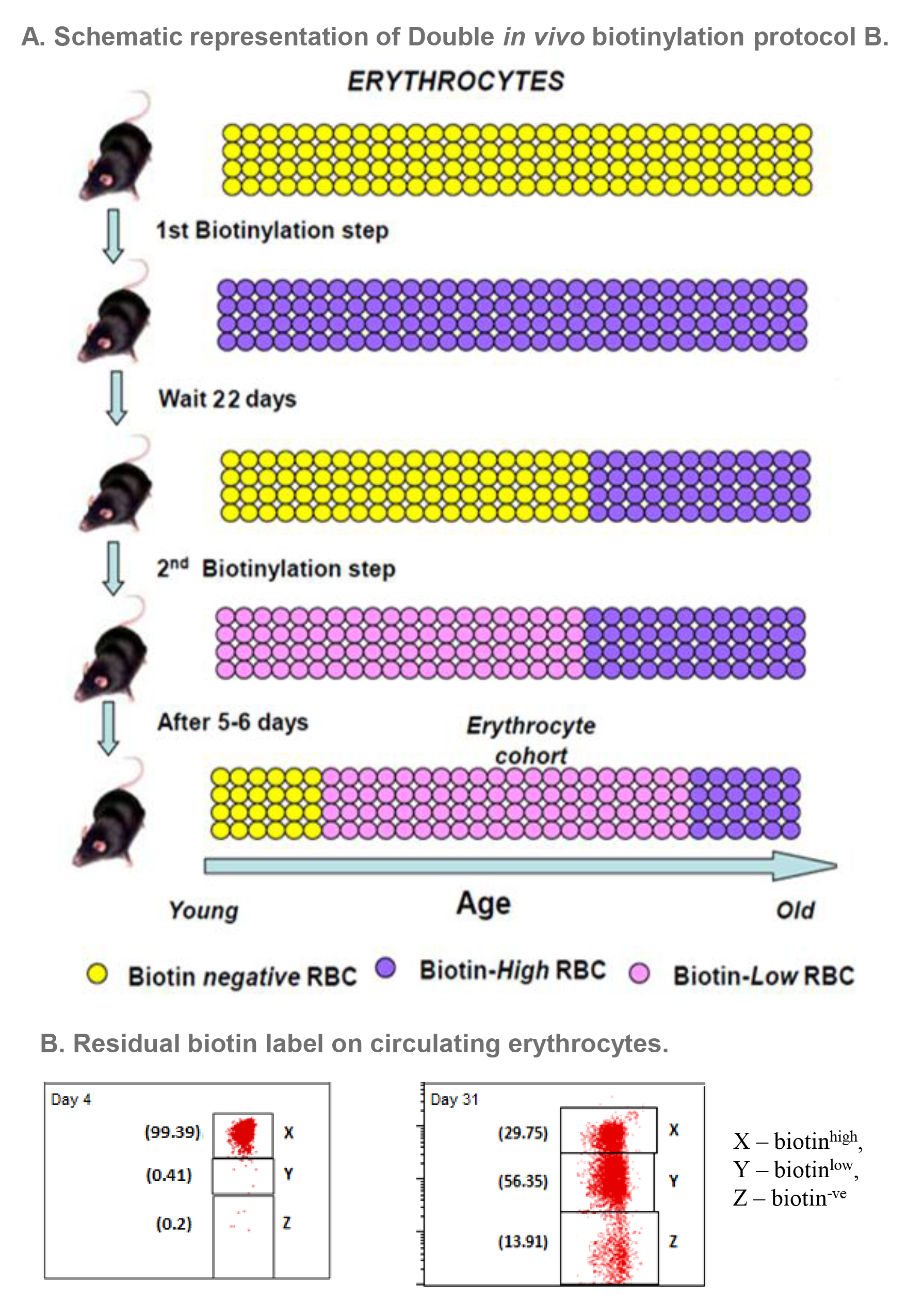

Supplement: S2 Fig — C57BL/6 mice were administered intravenously three daily doses of 1mg BXN (first biotinylation step). After a rest for 22 days, a single additional dose of 0.6mg BXN was administered (second biotinylation step). Blood was collected at different time points and distribution of biotin label on erythrocytes was examined by staining the cells with Streptavidin-APC followed by flow cytometry. The scheme of the experiment is given in panel A, and Biotin label on circulating erythrocytes at different time points is given in panel B. Erythrocyte populations in boxes X, Y and Z represent biotinhigh, biotinlow and biotinnegative populations of erythrocytes respectively; values in parentheses represent percentage of cells in different boxes. (TIF) [file pone.0132697.s002.tif]

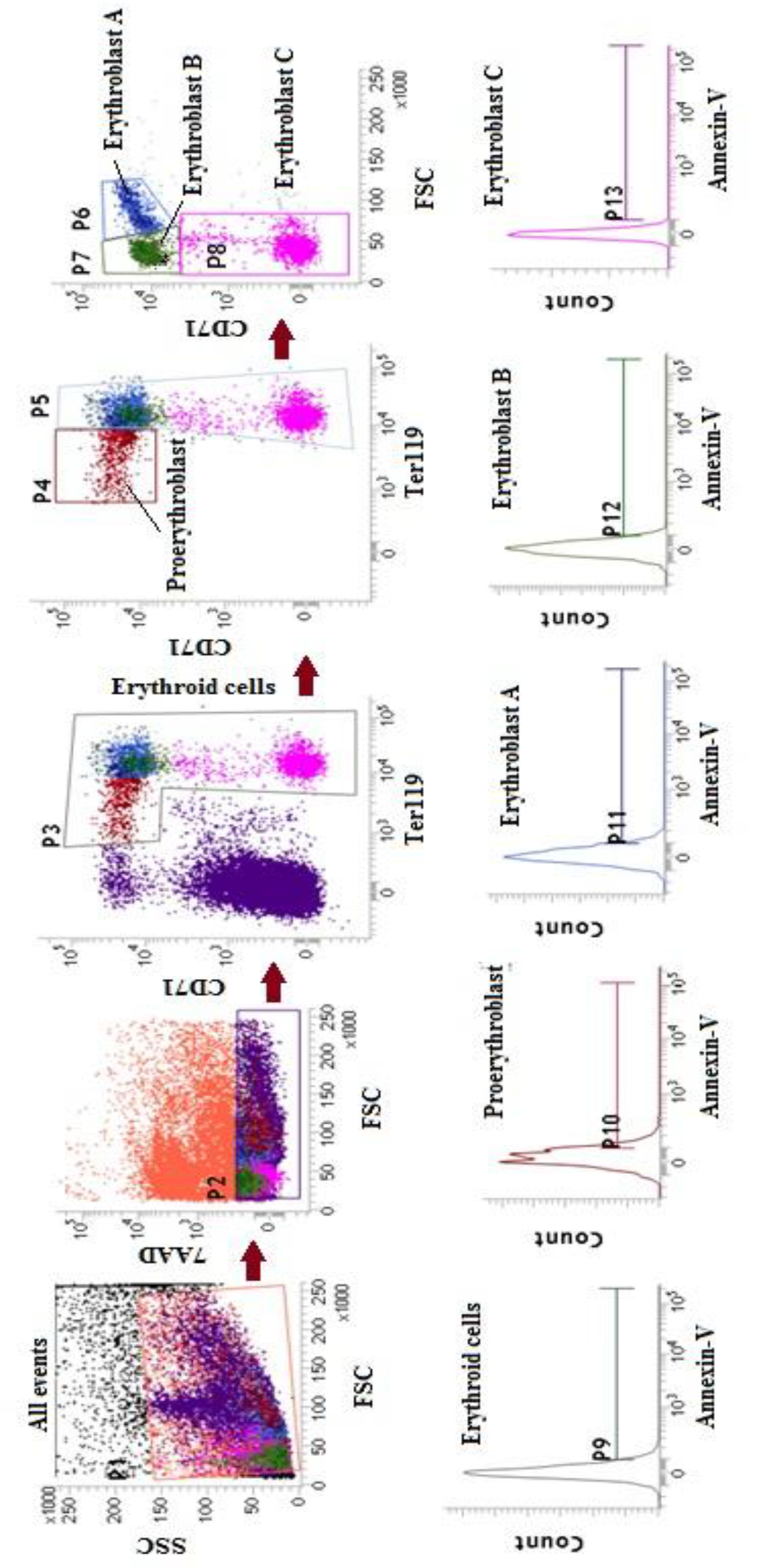

Supplement: S3 Fig — Mice were sacrificed and their femur and tibia-fibula dissected out. Bone marrow cells were isolated from these bones and single cell suspensions were prepared. 1x10^6^ cells from freshly prepared bone marrow suspensions were stained with Ter119-APC, CD71-FITC, Annexin-V-PE and 7AAD. A representative flow diagram from the bone marrow of a control mouse with all the gating strategies has been shown above. Bone marrow cells were gated on 7AAD- population and the erythroid cells were denoted as the Ter119+ population. The Ter119medCD71high population amongst these erythroid cells were identified as Proerythroblasts. The remaining Ter119+ erythroid cells were further delineated by relative CD71 expression and their FSC and the different stages of maturation were identified as: Erythroblasts A (Ter119highCD71highFSChigh), Erythroblasts B (Ter119highCD71highFSClow), and Erythroblasts C (Ter119highCD71lowFSClow). Annexin-V+ cells amongst each of these erythroid populations indicate the apoptotic cells of erythroid lineage. (TIF) [file pone.0132697.s003.tif]

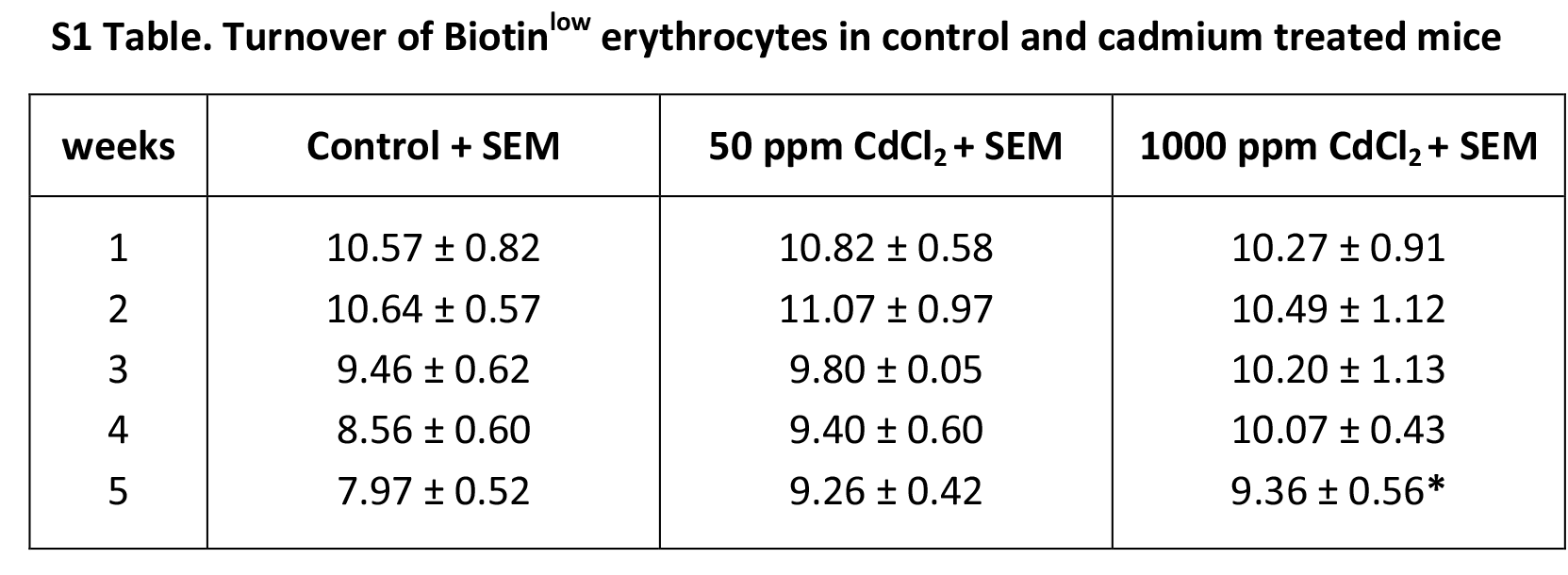

Supplement: S1 Table — Mice were given cadmium dissolved in drinking water (50 ppm and 1000 ppm). Mouse erythrocytes were labeled with biotin in vivo by the two step biotinylation procedure following DIB protocol A. At weekly intervals erythrocytes were stained ex vivo with Streptavidin-APC and proportions of the different age cohorts were determined. Turnover profile of biotinlow erythrocytes in mice exposed to 50 ppm and 1000 ppm of cadmium chloride is given in the above table. Each value represents mean ± SEM of observations on 10–15 mice. *p<0.05 for comparison of the groups. Statistical analysis was done using Student t-test. (TIF) [file pone.0132697.s004.tif]

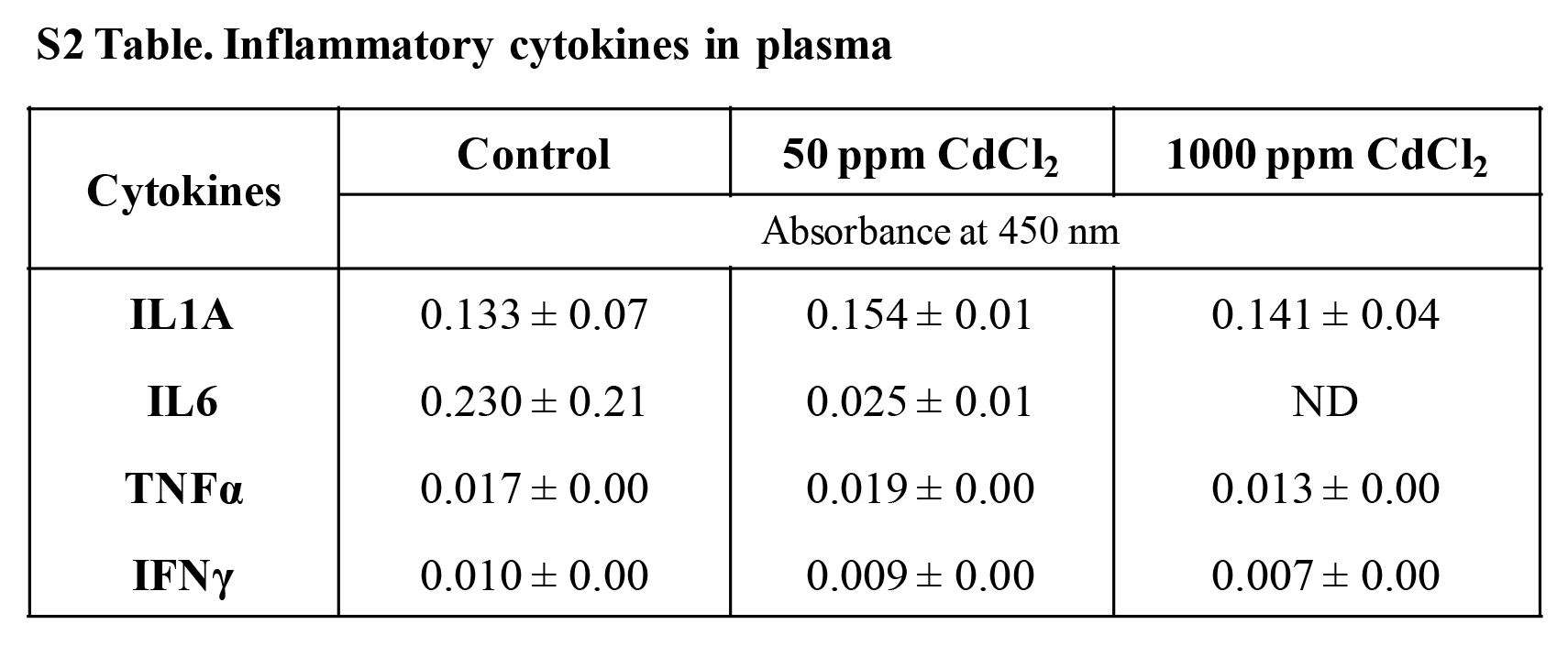

Supplement: S2 Table — Mice were given cadmium chloride dissolved in drinking water (50 ppm and 1000 ppm). After 5 weeks of exposure blood was collected by terminal bleeding and plasma was isolated. Presences of inflammatory cytokines such as IL1A, IL6, TNFα and IFNγ in plasma were detected by a Multi-Analyte ELISArray kit, and the results are given above. Each value represents mean ± SEM of observations on 5–6 mice. (TIF) [file pone.0132697.s005.tif]
